# Anthropogenic drought dominates groundwater depletion in Iran

**DOI:** 10.1038/s41598-021-88522-y

**Published:** 2021-04-28

**Authors:** Samaneh Ashraf, Ali Nazemi, Amir AghaKouchak

**Affiliations:** 1 Department of Building, Civil and Environmental Engineering, Concordia University, Montreal, Canada; 2 Department of Civil and Environmental Engineering, University of California, Irvine, CA USA

**Keywords:** Climate sciences, Environmental sciences, Hydrology

## Abstract

Using publicly-available average monthly groundwater level data in 478 sub-basins and 30 basins in Iran, we quantify country-wide groundwater depletion in Iran. Natural and anthropogenic elements affecting the dynamics of groundwater storage are taken into account and quantified during the period of 2002–2015. We estimate that the total groundwater depletion in Iran to be ~ 74 km^3^ during this period with highly localized and variable rates of change at basin and sub-basin scales. The impact of depletion in Iran’s groundwater reserves is already manifested by extreme overdrafts in ~ 77% of Iran’s land area, a growing soil salinity across the entire country, and increasing frequency and extent of land subsidence in Iran’s planes. While meteorological/hydrological droughts act as triggers and intensify the rate of depletion in country-wide groundwater storage, basin-scale groundwater depletions in Iran are mainly caused by extensive human water withdrawals. We warn that continuation of unsustainable groundwater management in Iran can lead to potentially irreversible impacts on land and environment, threatening country’s water, food, socio-economic security.

## Introduction

By suppling ~ 36% of drinking water and ~ 42% of agricultural water, groundwater is a key freshwater resource globally^1,2^. During the current state of “*Anthropocene*”, groundwater reserves are under enormous stress due to both natural and anthropogenic pressures^3,4^. Naturally, groundwater is sensitive to variability and change in hydroclimatic conditions^5–7^. For instance, increased evaporation due to a warmer climate reduces groundwater recharge^8^, which is also sensitive to landscape features, such as vegetation and soil characteristics^9,10^. In parallel, groundwater availability is also affected by human water withdrawals to support various socio-economic activities^11^. Uptakes from groundwater reserves have increased substantially in recent years due to ever-increasing global population and water use per capita^12^.

Despite current pressures on groundwater resources, they have a critical role in maintaining water security. Currently one third of the world’s population living in water-stressed regions^13^, particularly in semi-arid and arid regions of Asia, the Middle East and North Africa as well as the Mediterranean countries. In many parts of these regions, groundwater is the only reliable source of water; because surface water is seasonally or permanently absent^14^. As water demands in these regions are mainly concentrated around food productions—e.g., ~ 85% of water use in the Middle East is exclusively used for irrigation^15^—groundwater availability and food security become massively intertwined and are linked to national and regional security^16^. The availability of groundwater resources becomes more critical in a warmer and more populated world^17–19^, as surface water resources deplete even more under increasing temperature^20–22^, causing elevated competition over the remaining surface water resources^15,23–26^.

Although accurate monitoring of groundwater resources is essential for provision of effective management practices^27^, groundwater monitoring has not well-attended in developing countries^28^, mainly due to the hidden nature of groundwater and the lack of recognition for the human impacts on groundwater resources^29^. Data coming from the Gravity Recovery and Climate Experiment (GRACE) satellite has provided a grand opportunity for monitoring changes in groundwater storage and detecting depletion at larger scales^30–32^; however still the value of in-situ groundwater monitoring is unquestionable, particularly at smaller basin and sub-basin scales. Here, we focus on Iran, a country where natural dryness is mixed with rapid socio-economic development, growing water demand particularly for agriculture, and unsustainable land and water management^25,33^. We analyze the dynamics of Iran’s groundwater resources using the publicly-available data for average groundwater level at basin and sub-basin scales, published by Iran’s Ministry of Energy. The unique feature of our study is in quantifying the variation in groundwater storage and its dependencies with potential natural and anthropogenic drivers solely based on basin and sub-basin estimations for natural and anthropogenic drivers of groundwater dynamics, extracted from country-wide networks of in-situ observations. This allows extracting a set of ground truths for causes of groundwater depletion, and to determine the potential impacts of groundwater depletion on water, land and environment at basin and country-wide scales.

## Results

**Figure 1:**
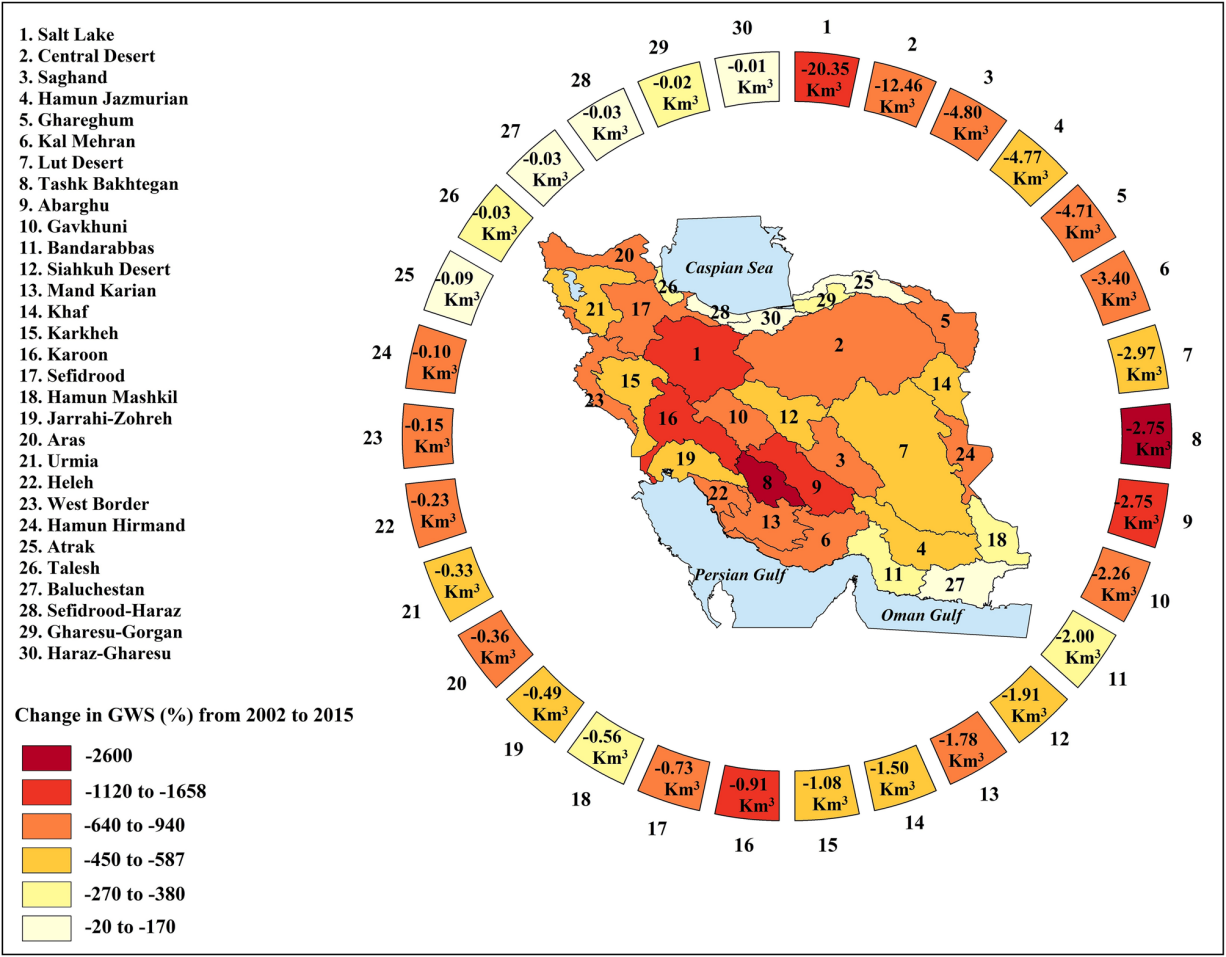
Groundwater depletion (in km^3^) during 2002–2015 across major basins in Iran. In the outer circle, basins are ordered based on their total depletion in groundwater storage in km^3^ from the largest to the smallest. Shades of color show relative changes in groundwater storage in % during the study period (This figure is created using ArcGIS 10.8).

Based on average estimated groundwater levels at the basin scales, Iran’s groundwater has been depleted around ~ 74 km^3^ during 2002–2015 (Fig. 1). This amount is ~ 1.6 times larger than the historical high storage in the Lake Urmia (~ 46 km^3^ in 1996)^34^, which is the largest lake in the Middle East and the sixth largest saltwater lake on Earth^35^. Although during the study period, there are limited sub-basins in which the groundwater storage has increased (see Figure S1 in Supplementary Information), all major basins experienced some degrees of groundwater depletion, with rates ranging from 20 to 2600% decline in the course of 14 years—see Fig. 1. The highest amount of depletion is observed in the Salt Lake basin (Basin 1 in Fig. 1), which supports more than 26% of Iran’s population (see Figure S2 in Supplementary Information) and is depleted ~ 20 km^3^ in the considered 14-year timeframe. This depletion is ~ 81% of the total depletion in the U.S. High Plains, the most depleted aquifer in the United States, during the most severe historical drought of 1976–1977^1^. Tashk Bakhtegan basin, supporting ~ 3.5% of Iran’s population, shows the highest relative change in groundwater storage (~ 2600% decrease). The lowest depletion (~ 0.01 km^3^) and the lowest relative change in groundwater storage (~ 20%) is observed in Haraz-Gharesu basin in the north of the country, serving ~ 4% of Iran’s total population. At the country-scale, Iran’s groundwater reserves undergo a progressive depletion with the rate of ~ 5.25 km^3^/y from 2002 to 2015, marking the total growth of ~ 1752% in 14 years (Figure S3 in Supplementary Information). This rate is ~ 92% of the long-term depletion rate in the U.S. High Plains during 1950–2007 (~ 5.7 km^3^/y)^1^.

Considering Fig. 1 and Figure S2 in the Supplementary Information, it is revealed that groundwater depletion is much more severe in populated basins in the West, Southwest and Northeast of Iran, where largest irrigated lands of wheat and barley, Iran’s two strategic crops, are located—see Figure S4 in Supplementary Information. For instance, Karkheh basin—the food basket of Iran and home to 9% of Iran’s total irrigated lands and ~ 11% of the country’s total wheat production^36^—has experienced the depletion rate of ~ 0.08 km^3^/y from 2002 to 2015 (~ 1.08 km^3^ depletion during 14 years; Basin 15 in Fig. 1). In addition, groundwater is a major supply for domestic and irrigated water demands in Karoon basin^37^, which is depleted with the rate of ~ 0.06 km^3^/y from 2002 to 2015 (~ 0.91 km^3^ total depletion in the considered timeframe—see Basin 16 in Fig. 1). Considering the current level of groundwater depletion and the growing scarcity in surface water resources in both basins^33^, there will be major concerns for maintaining irrigated agriculture and domestic water use in the years to come. The implication of this major groundwater depletion on regional and national water security in Iran is discussed in more details below.

**Figure 2:**
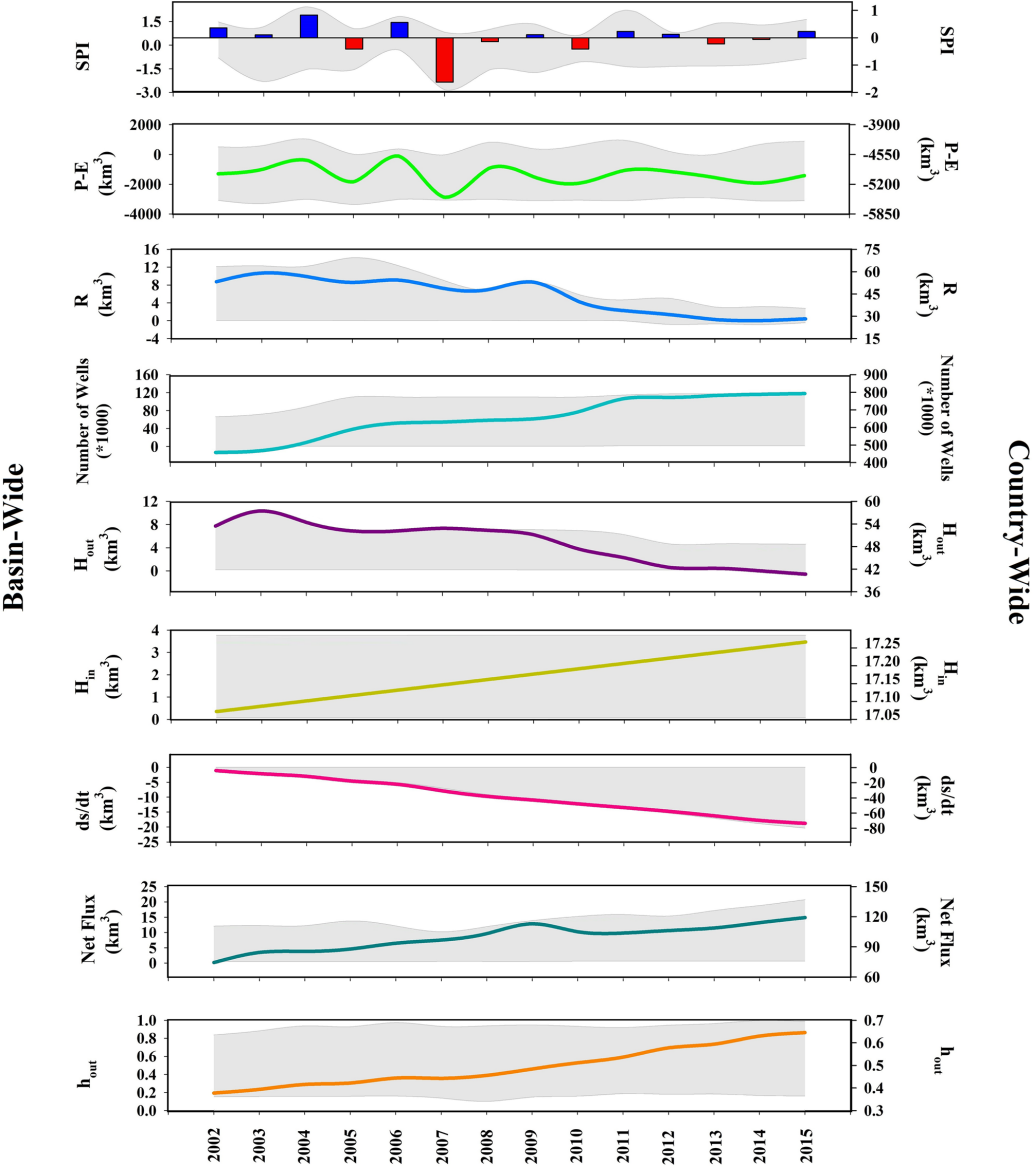
Evolution in groundwater storage (*ds/dt*) and its corresponding natural and anthropogenic drivers during 2002–2015 at the country (right y-axis) and basin scales (left y-axis). *SPI* is Standard Precipitation Index; *P* − *E* is precipitation minus pan evaporation; *R* is recharge, *H*_*out*_ is human outflow, *H*_*in*_ is human inflow, *R* + *H*_*in*_*-ds/dt* is the net outgoing flux and *h*_*out*_ is the normalized human outflow. Country- and basin-wide dynamics in each variable are shown by solid lines and gray envelope, respectively (This figure is created using R).

The changing dynamics of groundwater storage (*GWS*) is determined by the interplay between climatic, hydrologic and anthropogenic drivers. Figure S5 in the Supplementary Information shows the long-term annual averages of precipitation (*P*), pan evaporation (*E*), groundwater recharge (*R*), anthropogenic water withdrawals (i.e., human outflow; *H*_*out*_) and return flow to aquifers (i.e., human inflow; *H*_*in*_) during the considered timeframe of 2002–2015. At the country scale (Fig. 2), the significant decrease in *GWS* corresponds with decreasing surface water availability (*P* − *E*), which accordingly decreases recharge rates. For instance, during the severe drought of 2007, ~ 20% decrease in *P* − *E* in Iran has caused ~ 10% lower country-wide groundwater recharge, leading to a significant decline in the groundwater storage by ~ 40%, compared to 2006 (Figure S3 in Supplementary Information), while the increase in human water withdrawal was only ~ 1.3%. This vividly shows the important role of natural recharge in maintaining country-wide groundwater storage.

**Figure 3:**
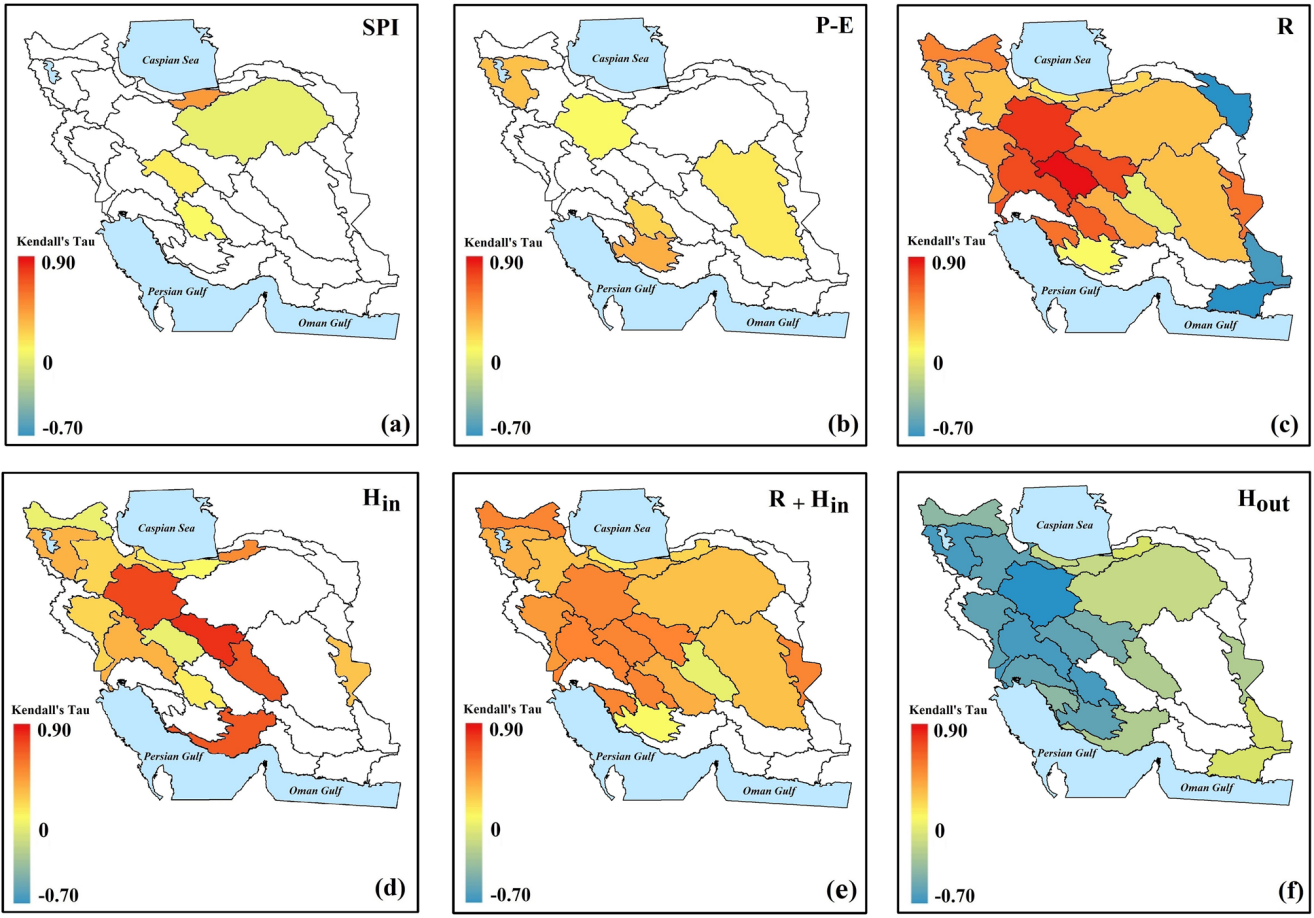
Dependency between annual changes in groundwater storage (*ds/dt*) and climatic, hydrologic and anthropogenic drivers of change across the 30 major Iranian basins during 2002–2015. In each panel, only basins with significant dependencies are shaded. Significant dependency is characterized by Kendall’s rank coefficients when *p* value ≤ 0.05 (This figure is created using ArcGIS 10.8).

By moving from the country-scale to the finer basin scale, however, the impact of water withdrawals becomes more apparent and dominates the climatic forcing and potential surface water availability, characterized by Standard Precipitation Index (*SPI*)^38^ and *P* − *E*, respectively. Based on Fig. 3a and b and according to Kendall’s rank coefficient^39^, only in limited numbers of basins in Iran, changes in groundwater storage (*ds/dt*) depend significantly (*p* value ≤ 0.05) on variations in *SPI* and *P* − *E* (4 and 5 basins out of 30 basins). In contrast, changes in groundwater storage (*ds/dt*) show significant positive dependence with recharge (*R*), human inflow (*H*_*in*_) and total inflow (i.e., *R* + *H*_*in*_) in ~ 63%, ~ 50% and ~ 63% of Iran’s basins respectively—see Fig. 3c–e. In addition, changes in groundwater storage (*ds/dt*) significantly depend on human outflow (*H*_*out*_) in 70% of Iran’s basins—see Fig. 3f. This highlight decreasing natural recharge (*R*) along with increasing net anthropogenic uptakes from aquifers (i.e., *H*_*out*_–*H*_*in*_) as two key drivers of massive decline in groundwater storage in Iran.

## Discussion

**Figure 4:**
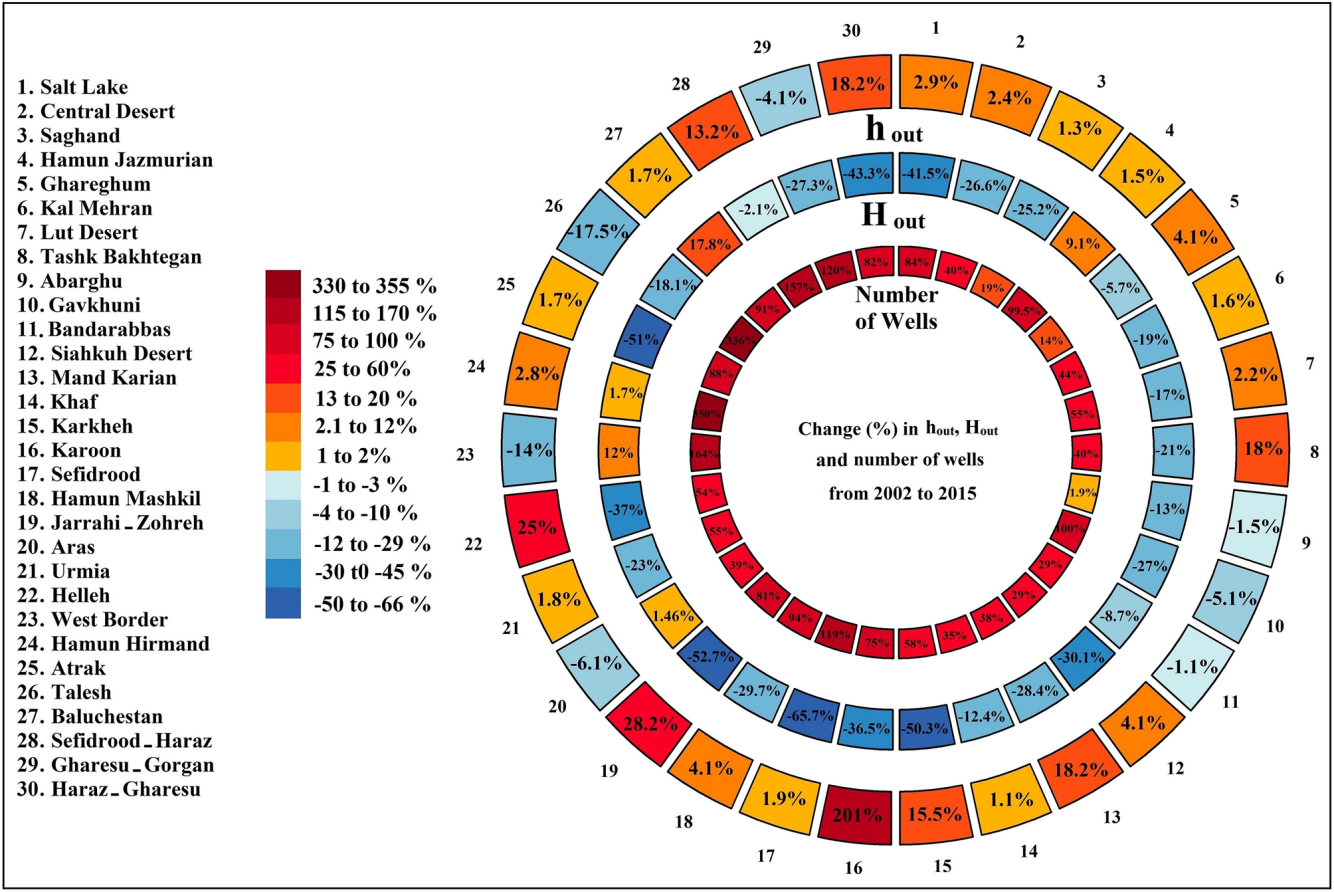
Relative changes in normalized human annual outflow (*h*_*out*_), along with relative changes in total human outflow (*H*_*out*_) and the number of wells from 2002 to 2015 across major Iranian basins, arranged in outer circle, middle circle and the inner circle, respectively. Basins are sorted with the same order configured in Fig. 1 (This figure is created using R).

Iran has been under a prolonged drying condition since the early twenty-first century, revealing itself by vanishing lakes and wetlands^40^ along with excessive water stress across the country^25^. As it was previously shown for the case of surface water availability in Iran^33^, such drought conditions are human-induced and are exemplifiers of “anthropogenic droughts”^41,42^, which refer to water stress caused or intensified by aggressive, short-sighted and unsustainable land and water management^25,41–43^. Here, the condition of anthropogenic drought is manifested by excessive ratios of water withdrawal to available renewable groundwater resources across Iran’s major basins. This is not counter intuitive: the number of registered wells has increased from ~ 460,000 in 2002 to ~ 794,000 in 2015 (see Fig. 4; inner circle), with increasing rate across all basins in Iran, ranging from 1.9% in Abarghu to 350% in Hamun Hirmand—see also Figure S7 in Supplementary Information. Despite the increasing change in the number of wells, anthropogenic groundwater withdrawals (*H*_*out*_) have decreased in 25 out of the 30 basins during the considered study period—see Fig. 4 (middle circle) and Figure S7 in the Supplementary Information. We argue that the decreasing change in the basin-scale *H*_*out*_—also can be seen in Fig. 2 at the country scale—is a clear testament for overexploitation of groundwater reserves rather than regulation of groundwater uptakes. To illustrate this, we calculate the normalized human outflow (*h*_*out*_) as the ratio of withdrawal (*H*_*out*_) to total net outgoing flux (*R* + *H*_*in*_ − *ds/dt*) at each major basin. This ratio indicates the proportion of human water withdrawal from the total outgoing flux at the basin scale that includes all other drivers that can potentially contribute to the decline in the of groundwater storage. As shown, *h*_*out*_ has increased in 23 out of 30 basins throughout the country—see Fig. 4 (outer circle). The overexploitation of Iran’s groundwater reserves become more apparent, by knowing that in half of its basins, the average *h*_*out*_ stays above 0.9 throughout the study period, revealing that more than 90% of the total outgoing flux from basin-scale groundwater reserve is solely due to human uptakes—see Figure S7 in the Supplementary Information. Increasing normalized human outflow (*h*_*out*_) but decreasing total withdrawal (*H*_*out*_) indicates that in some basins, groundwater reserves may have reached a critical tipping point where water withdrawals are limited by depleted groundwater reserves. As it is clear from Figure S8 in the Supplementary Information, the net outgoing flux is decreased in most of the country’s land, particularly in highly irrigated and/or populated regions despite substantial increases in normalized human outflows—see Fig. 4 (outer circle) as well as Figure S7 in the Supplementary Information.

Although the extensive groundwater depletion in Iran is largely anthropogenic, it has been exacerbated by meteorological and/or hydrologic droughts. Looking at the simultaneous country-wide variations in natural and anthropogenic drivers of change in groundwater storage (see Fig. 2), we argue that the groundwater response to human pressures has undergone three phases of evolution. These periods correspond to the wet years of 2002–2006 (positive *SPI*), dry years of 2007–2010 (negative *SPI*) and normal years of 2011–2015 (near zero *SPI*). During the first period, wet meteorological condition results in relatively higher effective precipitation and recharge. While number of wells substantially increased between 2002 and 2006 and withdrawals stood on its highest mean, accumulated groundwater depletion was the lowest compared to the other two periods. This 5-year wet period was substituted by a 4-year meteorological drought that increased dryness (i.e. decreased *P* − *E*), but did not largely affect recharge as well as withdrawals. After the dry year of 2010, despite increment in the number of wells and accordingly normalized water withdrawal, both recharge and withdrawals have decreased substantially. This reveals severe pressures on Iran’s groundwater reserves as a result of extremely aggressive human water uptakes in previous years.

Although the very recent wet period of 2019–2020 may have eased the groundwater stress in some parts of Iran^44^, our analysis clearly shows that Iran’s groundwater resources have depleted much faster than they have replenished. During the considered 14-year period, there are 12 out of 30 basins that experienced a continuously progressive depletion without a single year recovery. These basins are located mainly in the harvested areas of the country (Figure S4 in in the Supplementary Information) that are largely irrigated by groundwater resources. This is again not counter-intuitive: the total area equipped for irrigation in Iran during 2005–2010 was ~ 8.85 million ha, 62% of which is irrigated by groundwater (Table S1 in the Supplementary Information). In addition, the total irrigation area, as well as the area allocated for irrigation of Iran’s strategic crops (i.e., wheat and barley) have increased by ~ 12% and ~ 16% respectively during the study period (Figure S9 in the in the Supplementary Information). The irrigate areas for these two strategic crops declined after the dry year of 2010 but have been on the path of recovery since 2011 despite decline in total withdrawals. This is due to the fact that normalized withdrawals have increased due to growing number of wells, causing more stress on groundwater resources.

**Figure 5:**
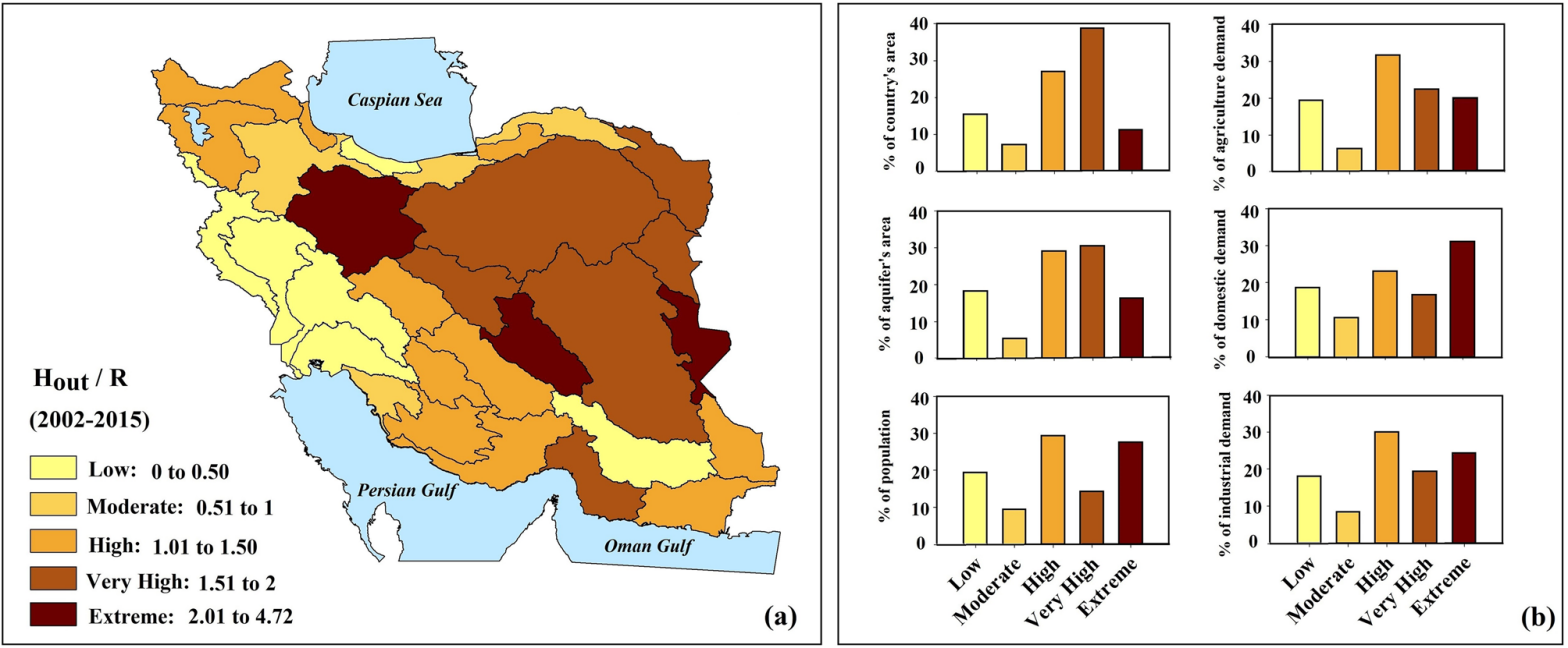
(**a**) The rate of groundwater overdraft during 2002–2015 in Iran; and (**b**) the percentage of population, land area, aquifer area and water demands in each overdraft category (This figure is created using ArcGIS 10.8).

Iran has long-standing issues with the inefficiency of its water distribution network, particularly in the agricultural sector^40,45^. We argue that attention to food production without improving the irrigation efficiency is a major cause for excessive groundwater overdraft in Iran^16,40^. Here we define excessive aquifer overdraft as a condition in which the total human water withdrawn exceeds the natural recharge rate over a given period. Groundwater overdraft marks a condition in which the groundwater reserves are unable to fully recover even in hydrologically wet years^25^. Figure 5 clearly reveals that around ~ 76% of Iran’s aquifer area (~ 77% of the country’s total area) is under excessive overdraft. If not reversed, this can cause major consequences in a way that threatens socio-economic and environmental security of Iran as a whole.

The first victim of Iran’s major groundwater depletion would be the country’s own food security. Irrigation includes more than 90% of Iran’s water withdrawal (Figure S11 in the Supplementary Information). The areas under excessive groundwater overdraft reside ~ 71% of Iran’s total population and include ~ 70% of the country’s total water demand—see Fig. 5b. Even in basins that are not currently categorized as areas with excessive overdraft, the agricultural water demand is high, and the normalized withdrawal is increasing rapidly (see Figure S8 in the Supplementary Information). We argue that growing overdraft will be expected in these basins, if the number of wells, irrigated areas and normalized withdrawals continue to increase.

**Figure 6:**
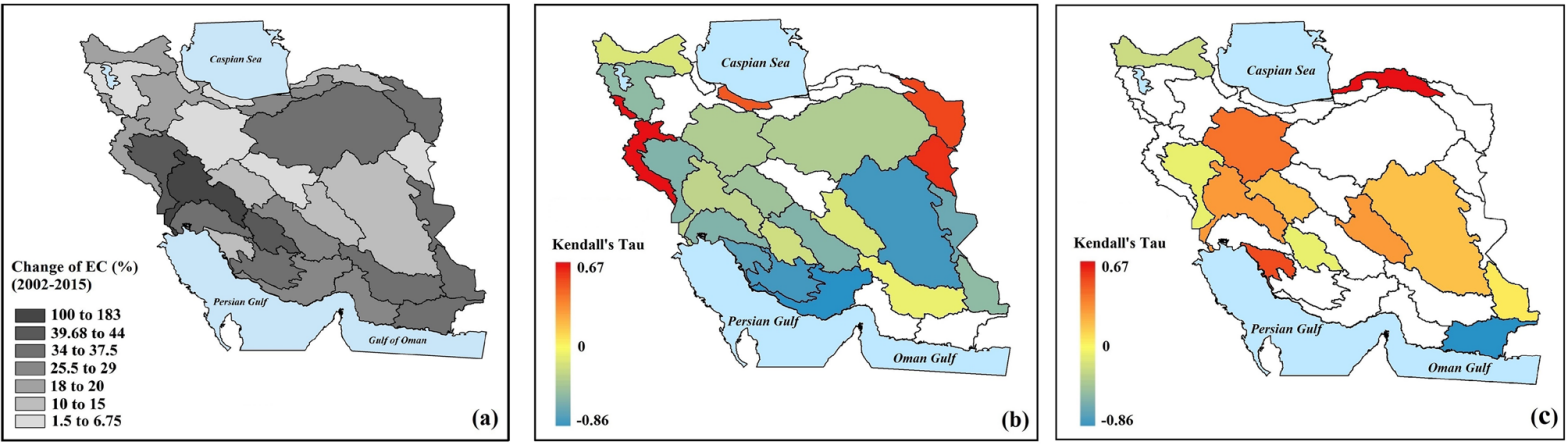
(**a**) Percent change in electrical conductivity (EC) during 2002–2015 across the 30 major basins in Iran and its significant dependence with basin-scale (**b**) changes in groundwater storage (*GWS*), and (**c**) human withdrawals (*H*_*out*_). Significant dependency is characterized by Kendall’s rank coefficients when *p* value ≤ 0.05 (This figure is created using ArcGIS 10.8).

The adverse impact of groundwater depletion can expand into other elements of environment. One major impact of groundwater depletion can be substantial increases in soil and groundwater salinity. Electrical Conductivity (EC) is a proxy for groundwater salinity assessments that is widely used for evaluating the quality of drinking and irrigation water. Figure 6a clearly shows a consistent increase in EC throughout the entire country during the study period. The rate of increase in salinity can be subject to spatial variability, with rates varying from ~ 1.5 to ~ 183% at the basin scale. Salinity negatively affects soil fertility, which can have devastating impacts on key food-producing regions in Iran (Figure S4 in Supplementary Information) and endangers long-term food security. We warn that this phenomenon is notable in some strategic regions and immediate actions are required to reverse this trend. For instance, Karkheh (basin number 15 in Fig. 1), the food basket of Iran, shows ~ 85% increase in EC during the considered 14-year timeframe. Additionally, in a place like Karoon basin (number 16 in Fig. 1) that shows the highest change in EC among all basins in Iran (~ 183%; see Fig. 6a), the groundwater is the water supply to 16 cities and several villages^37^. The analysis presented in Fig. 6b based on Kendall’s rank coefficient clearly shows that in 18 out of 30 major basins in Iran, increment in EC corresponds significantly with decrement in groundwater storage (*p* value ≤ 0.05). Furthermore, in these basins, the increase in EC significantly depends (*p* value ≤ 0.05) on increase in human water withdrawals (*H*_*out*_)—see Fig. 6c, revealing how excessive withdrawals increases EC through decreasing groundwater storage.

Another consequence of groundwater depletion is land subsidence. At least 25% of Iran’s population are living where the subsidence has the potential to reach to at least one meter within just a few years due to dramatically shrinking groundwater reserves^46^. This has been well documented in several plains within the Salt Lake basin^47,48^, the most depleted basin among the 30 major basins of Iran (see Fig. 1), as well as the western provinces of Iran, with the highest rate of subsidence being ~ 18.9 cm per year as of 2019 (https://www.ncc.gov.ir/en/). Such an intense land subsidence can changes surface and sub-surface flow paths and cause major and irreversible decline in aquifer capacity^49,50^. This becomes even more worrisome by considering that regions with substantial rates of land subsidence are home to large communities, such as Tehran, Iran's capital and the most populated city in western Asia with the total population of 15 million. Tehran is vulnerable to seismic hazards due to high potential for tectonic activities. If significant decline in soil stability due to land subsidence caused by extreme groundwater overdraft^46^ is compounded with a major tectonic activity, it can potentially intensify earthquake impacts causing a tragic human catastrophe.

## Conclusion and outlook

Iran, with population of ~ 84 million, is ranked first in the Middle East in terms of total human water withdrawals and is responsible for ~ 34% of the total water withdrawal in the region^45^. Groundwater is the main water source in Iran, accounting for almost 60% of Iran’s freshwater uptakes and therefore has a key role in maintaining national water security^45^. Using published data of average groundwater level along with natural recharge, human withdrawals and return flows, along with precipitation and evaporation at basin and sub-basin scales, we assess the compounding effects of climatic, hydrologic and anthropogenic drivers on the dynamics of groundwater storage in Iran during 2002–2015. Results illustrate that a severe anthropogenic drought, caused by extensive groundwater withdrawals, threatens groundwater sustainability in Iran. This is particularly the case in highly irrigated and populated regions in northwest, west and northeast of the country, where water demand drastically exceeds natural renewable water supply. Our finding shows that ~ 77% of Iran’s land (i.e., 23 out of 30 basins) is under extreme groundwater overdraft, where the rate of human uptake is more than three times higher than the rate of natural recharge. This has led to significant groundwater depletion, manifested by dried up wells across the country. Water scarcity can be a devastating constraint to food security in Iran, particularly in light of the calorie requirements for a nation that is currently under harsh international sanctions and deals with various socio-economic, environmental and geopolitical tensions.

As we noted, impacts of groundwater depletion in Iran are not only limited to water and food security and have already transcended to other elements of environment and caused a country-wide increase in soil salinity and increasing intensity and frequency of land subsidence. Land subsidence due to extensive groundwater withdrawal can consequently reduce aquifer capacity and therefore groundwater availability. In the absence of effective management practices, which is regretfully the case, this inevitably intensifies competition over limiting groundwater resources by more aggressive groundwater withdrawal, which in turn causes more land subsidence. This forms a vicious feedback process that is still not well understood. Although we are not able to characterize this important impact due to mismatch in the temporal and spatial characteristics of data related to groundwater storage and land subsidence, we highly encourage future research in this regard. In addition, land subsidence due to excessive groundwater withdrawal decreases soil stability, which is an important concern in highly populated areas—like Tehran, Iran’s capital—that are also prone to seismic activities. We hope these lines can attract the attention of decision makers in Iran.

Due to a significant decrease in surface runoff, protecting the existing groundwater resources in Iran is deemed important for facing the ever-growing water demand. This requires integrated management strategies for balancing water supply and demand at the basin and sub-basin scales. For a country like Iran with awfully low irrigation efficiency^45^, immediate actions are needed to improve water use. We believe that this is the key to the environmental security of Iran that will be likely under more stress due to prolonged natural and anthropogenic droughts, associated with heightened climate variability and change.

## Methods

### Study area and available data

Iran has the area of 1,648,195 km^2^, marking the country the second largest country in the Middle East and the 18th largest country in the world. Iran’s surface water resources is limited. More than 99% of the Iran’s land is covered by soil and vegetation. Geographical distribution of surface water supplies and water demands are extremely heterogeneous. Iran has a diverse climate. Having 35.5% of its land in hyper-arid, 29.2% in arid, 20.1% in semiarid, 5% in Mediterranean, and the rest in humid and hyper-humid climate, the country as a whole is characterized by an arid/semi-arid climate. The annual precipitation ranges from less than 50 mm in the southeast and central parts to more than 1600 mm in some coastal regions near the Caspian Sea. On the other hand, pan evaporation estimates are ranging between 1500 and 2000 mm. On average, Iran’s precipitation is less than one-third of the global average, while its evaporation is more than three times of the global average^45^.

Iran includes 30 major basin and 609 sub basins. There are no aquifers in 72 sub-basins and 59 sub-basins are not equipped with groundwater monitoring wells by the end of 2016. The average monthly water level data for the remaining 478 sub-basins, and across the 30 major basins in Iran during the period 2002–2015 have been published by Iran’s Ministry of Energy. These data considers in-situ data from Iran’s national groundwater monitoring network, comprising ~ 11,000 monitoring wells—see Figure S12 in Supplementary Information for the density of wells in each basin. Accordingly, monthly groundwater storage data (km^3^) at sub-basin and basin scales are estimated by multiplying the average change in groundwater level, aquifer area, and storage coefficient. Apart from groundwater level and storage, Iran’s Ministry of Energy provides monthly average precipitation and evaporation across these basins and sub-basins, obtained from a national weather network, measuring daily precipitation and Class A-pan evaporation in 4004 and 1730 in-situ stations across the country, respectively—see Figure S13 in Supplementary Information for the density of these stations in each basin. Iran’s Ministry of Energy also provides annual data for other components of groundwater balance, including groundwater recharge (*R*), human withdrawals (*H*_*out*_) and human return flow (*H*_*in*_), and Electrical Conductivity (EC) in all considered basins and sub-basins during 2002–2015. These data along many other information are publicly available and can be accessed at http://wrs.wrm.ir/amar/register.asp. The specific data used in this study are provided as Electronic Supplementary Information.

### Analysis of natural and anthropogenic drivers of groundwater depletion

We use annual Standard Precipitation Index (*SPI*)^38^, the most widely used index for monitoring climatic droughts, for understanding the magnitude and duration of meteorological droughts at the country, basin and sub-basin scales during the timeframe of 2002–2015. We use the difference between annual precipitation and pan evaporation (*P* − *E*) to quantify water availability and accordantly potential for groundwater recharge at the basin and sub-basin scales. For quantifying the dynamics of Groundwater Storage (*GWS*) at the basin scale, we use the aquifers’ water budget equation including natural and anthropogenic contributions formed by both withdrawals (*H*_*out*_) and return flows (*H*_*in*_) as the following^51^:

1 $$F_{n} = R + H_{in} - ds{/}dt = D + H_{out}$$

where *F*_*n*_ is the total outgoing flux, or the net flux, *R* is the natural recharge to the aquifer from surface water and adjacent aquifers; *D* is total natural discharge from the aquifer to the surface water and adjacent aquifers, as well as evaporative losses from shallow groundwater reserves. $ds{/}dt$ is the change in storage estimated using the observed groundwater monitoring wells. Accordingly, we define the basin-scale human withdrawal (*H*_*out*_), hereafter total withdrawal, as the ratio of water withdrawal to the total outgoing flux:

2 $$h_{out} = H_{out} {/}F_{n}$$

We use Kendall’s rank coefficient^43^, a non-parametric dependence measure, to inspect whether change in groundwater storage is dependent on changes in natural (*SPI*, *P* − *E*, *R*) and anthropogenic drivers (*H*_*in*_ and *H*_*out*_). The same methodology is used to address the dependency between *EC* and *GWS* as well as *EC* and *H*_*out*_.

## Supplementary Information

Supplementary Information 1.

Supplementary Information 2.
