# Supplementary material for: Anthropogenic drought dominates groundwater depletion in Iran
Source: Sci Rep. 2021 Apr 28;11:9135. doi: 10.1038/s41598-021-88522-y (PMC8080627; doi:10.1038/s41598-021-88522-y)
Supplement: Supplementary file 2 — Supplementary Information 2. [file 41598_2021_88522_MOESM2_ESM.docx]

**Supplementary Information for:**

**Anthropogenic Drought Dominates Groundwater Depletion in Iran**

**Samaneh Ashraf ^1^, Ali Nazemi ^1^, Amir AghaKouchak ^2^**

^1^ Department of Building, Civil and Environmental Engineering, Concordia University, Montreal, Canada

^2^ Department of Civil and Environmental Engineering, University of California Irvine, California, USA

Corresponding author: Samaneh Ashraf (samaneh.ashraf@mail.concordia.ca)


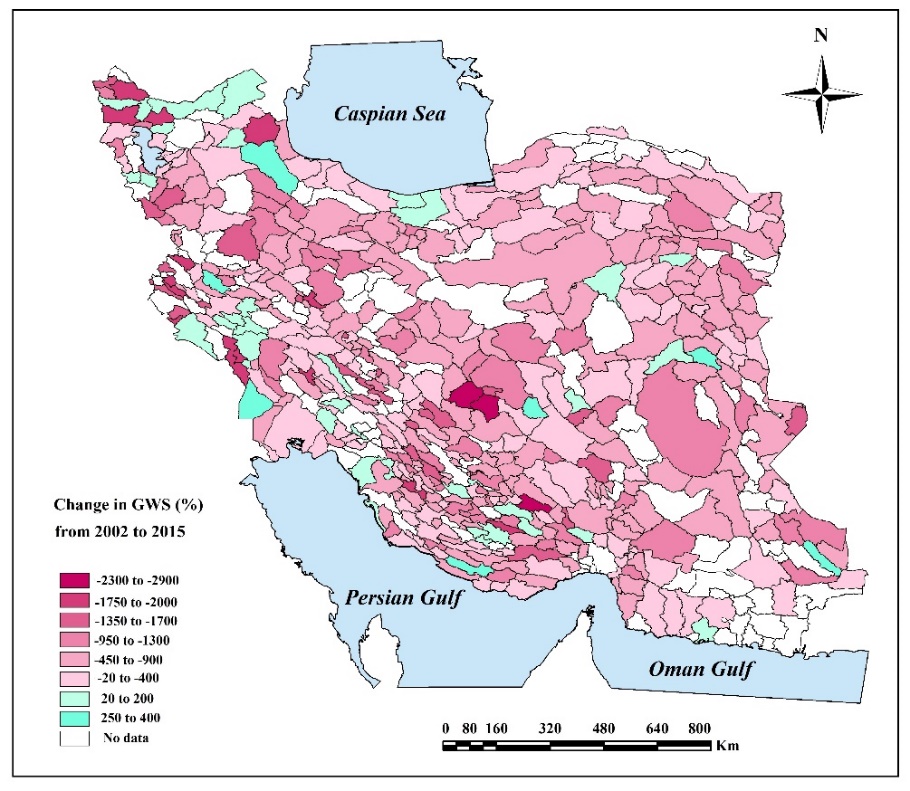


Figure S1. Percentage of change in Iran’s groundwater storage from 2002 to 2015 at the sub-basin scale (This figure is created using ArcGIS 10.8).


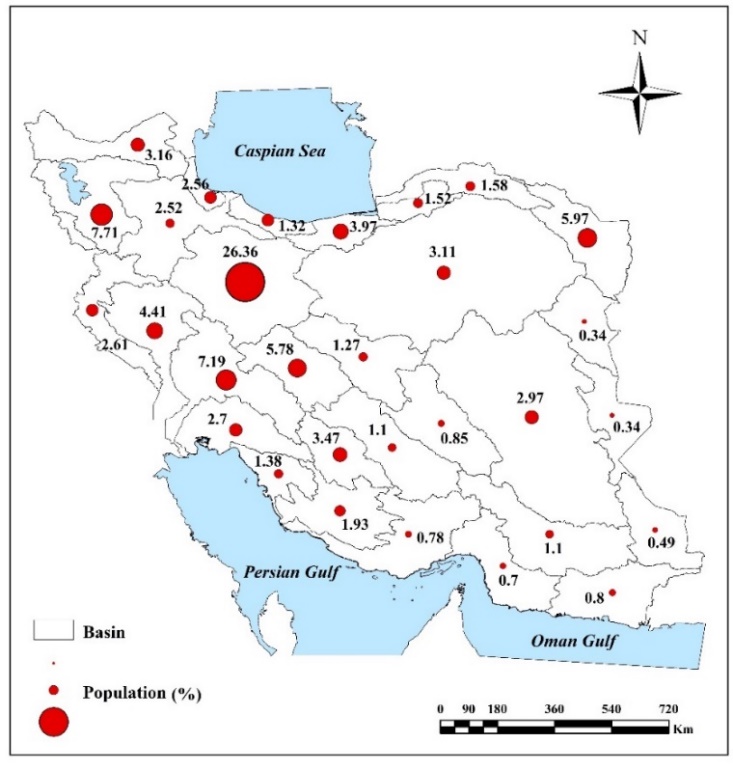


Figure S2. Ratio of Iran’s total population distributed in each major basin based on the census data of 2015 (Source of data: Statistical Center of Iran: <https://www.amar.org.ir/english>). (This figure is created using ArcGIS 10.8).


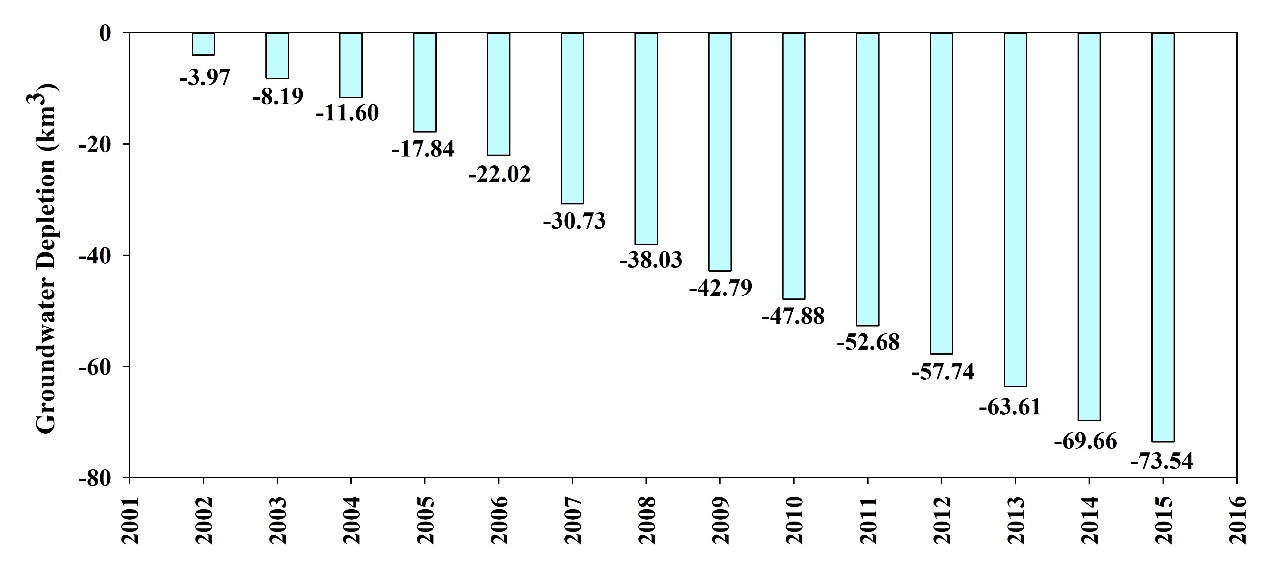


Figure S3. Progression in the country-wide groundwater depletion in Iran from 2002 to 2015 (This figure is created using R).


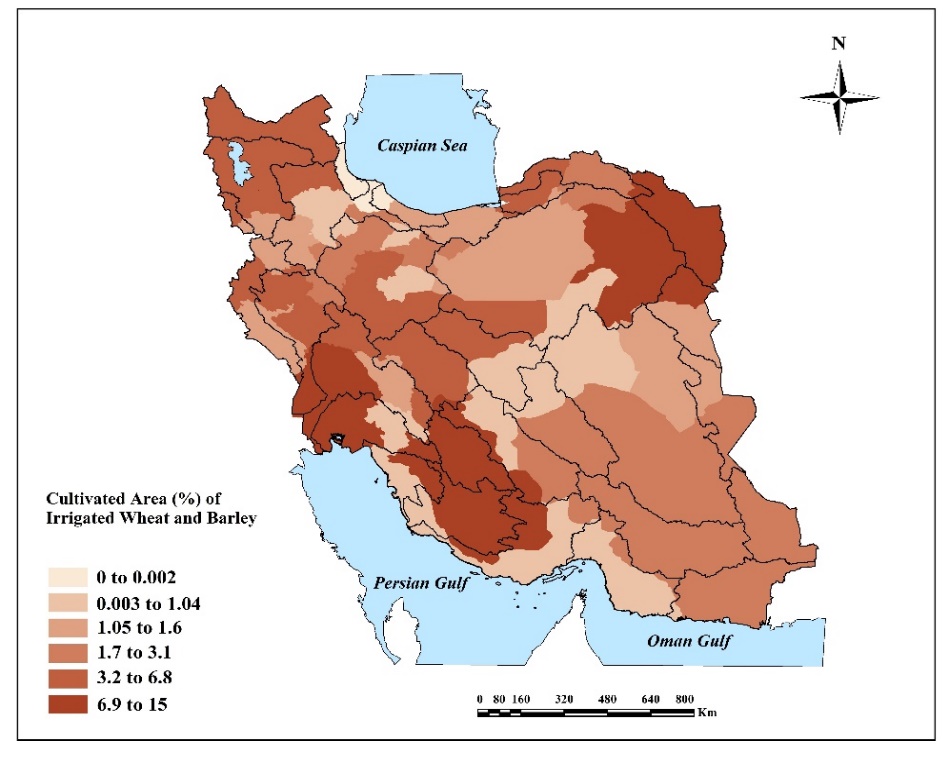


Figure S4. Iran’s irrigated wheat and barley farmlands (Source of data: Iran’s Ministry of Agriculture Jihad: [https://www.maj.ir/Index.aspx?page_=form&lang=1&PageID=11583&tempname=amar&sub=65&methodName=ShowModuleContent#](https://www.maj.ir/Index.aspx?page_=form&lang=1&PageID=11583&tempname=amar&sub=65&methodName=ShowModuleContent)) (This figure is created using ArcGIS 10.8).


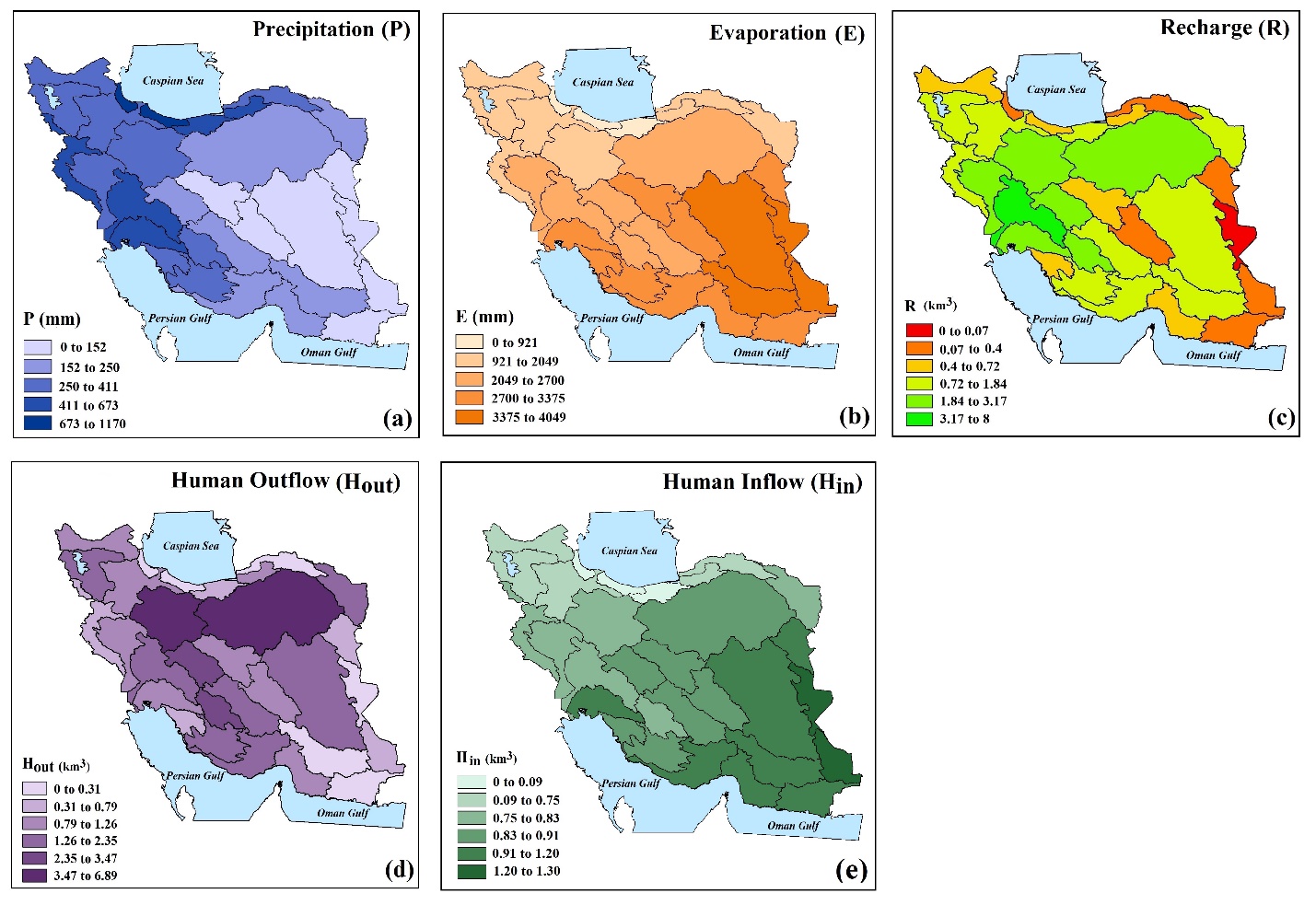


Figure S5. Mean annual (a) precipitation, (b) evaporation, (c) recharge, (d) human withdrawals and (e) human inflow across major Iranian basins during 2002-2015 (This figure is created using ArcGIS 10.8).


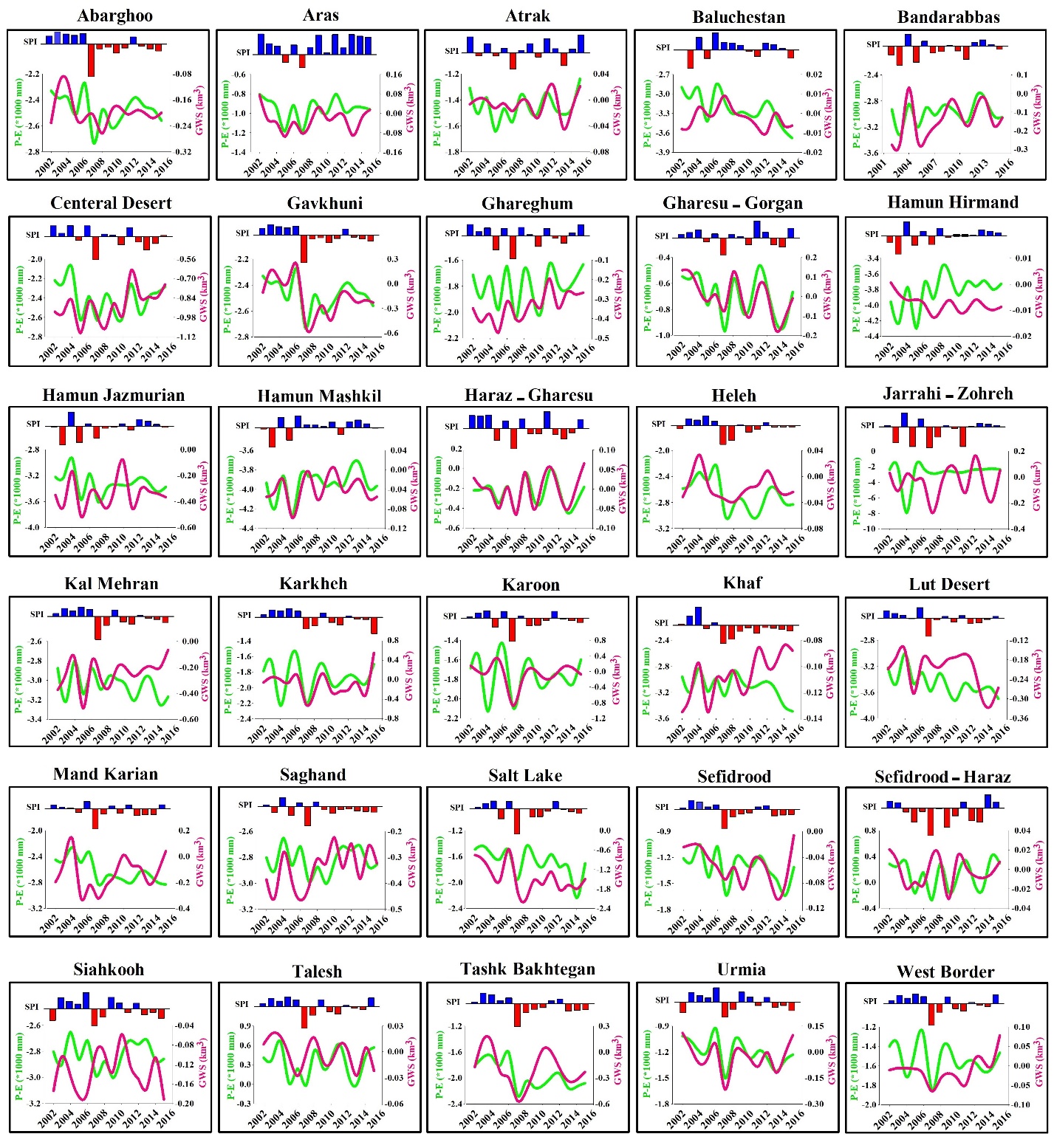


Figure S6. Changes in annual groundwater storage during 2002 to 2015 (purple lines) along with changes in SPI (bars) and *P-E* (green line) across thirty major basins of Iran (This figure is created using R).


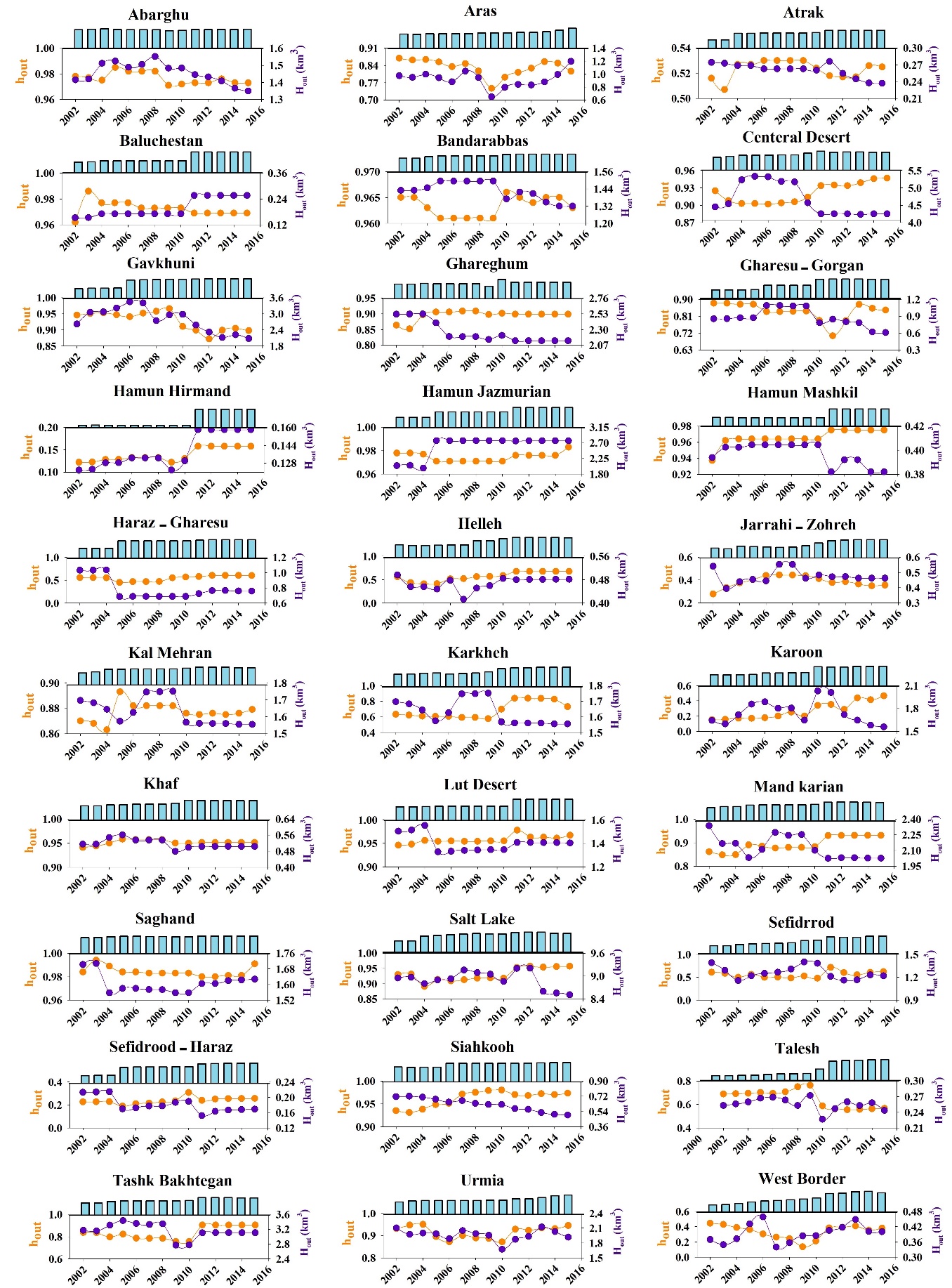


Figure S7. Number of exploitation wells (bars), total human groundwater withdrawal (H_out_) and normalized human outflow (h_out_) across the thirty major basins of Iran during 2002 to 2015 (This figure is created using R).


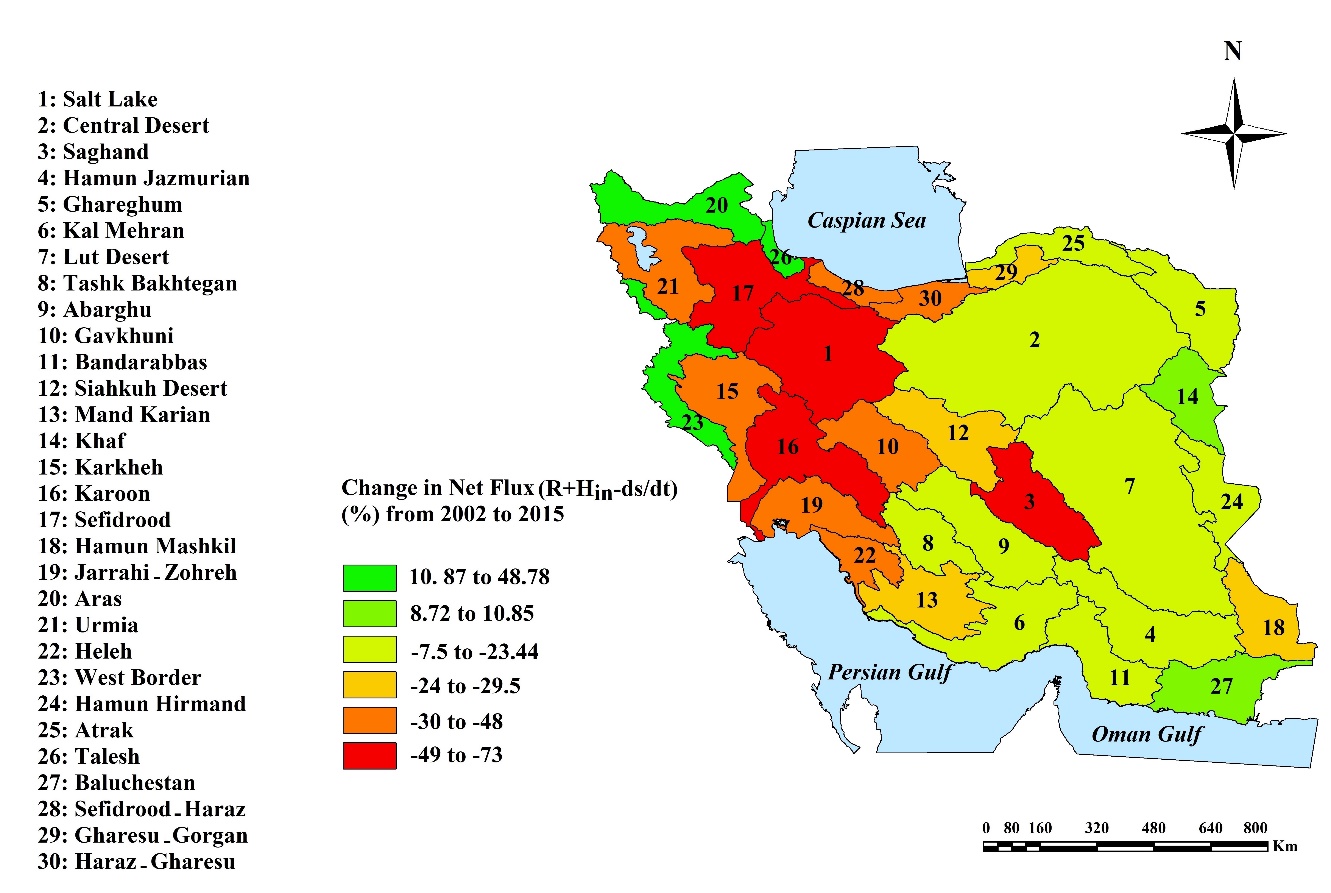


Figure S8. Percentage of change in groundwater net flux across Iran’s major basins during 2002 to 2015 (This figure is created using ArcGIS 10.8).

Table S1. The average area equipped for irrigation in Iran during 2005-2010 (Source of data: FAO's Global Information System on Water and Agriculture: [**h**ttp://www.fao.org/aquastat/en/geospatial-information/global-maps-irrigated-areas/irrigation-by-country/country/IRN](http://www.fao.org/aquastat/en/geospatial-information/global-maps-irrigated-areas/irrigation-by-country/country/IRN))

| **Region** | **Area equipped for irrigation (ha)** | | |
| --- | --- | --- | --- |
|  | **total** | **with groundwater** | **with surface water** |
| I (North Coastal) | 1 019 419 | 345 818 | 673 602 |
| II (Azerbaijan) | 963 735 | 316 382 | 647 353 |
| III (Khuzestan - West border) | 1 688 980 | 351 165 | 1 337 815 |
| IV (Fars - South Coastal) | 1 002 648 | 811 920 | 190 729 |
| V (Kerman - Sistan - Baluchistan) | 1 028 577 | 877 939 | 150 639 |
| VI (Esfahan) | 333 650 | 313 994 | 19 655 |
| VII (Central) | 1 189 844 | 1 093 710 | 96 135 |
| VIII (Khorasan) | 1 620 964 | 1 383 569 | 237 396 |
| **Iran (Islamic Republic of) total** | **8 847 818** | **5 494 495** | **3 353 323** |


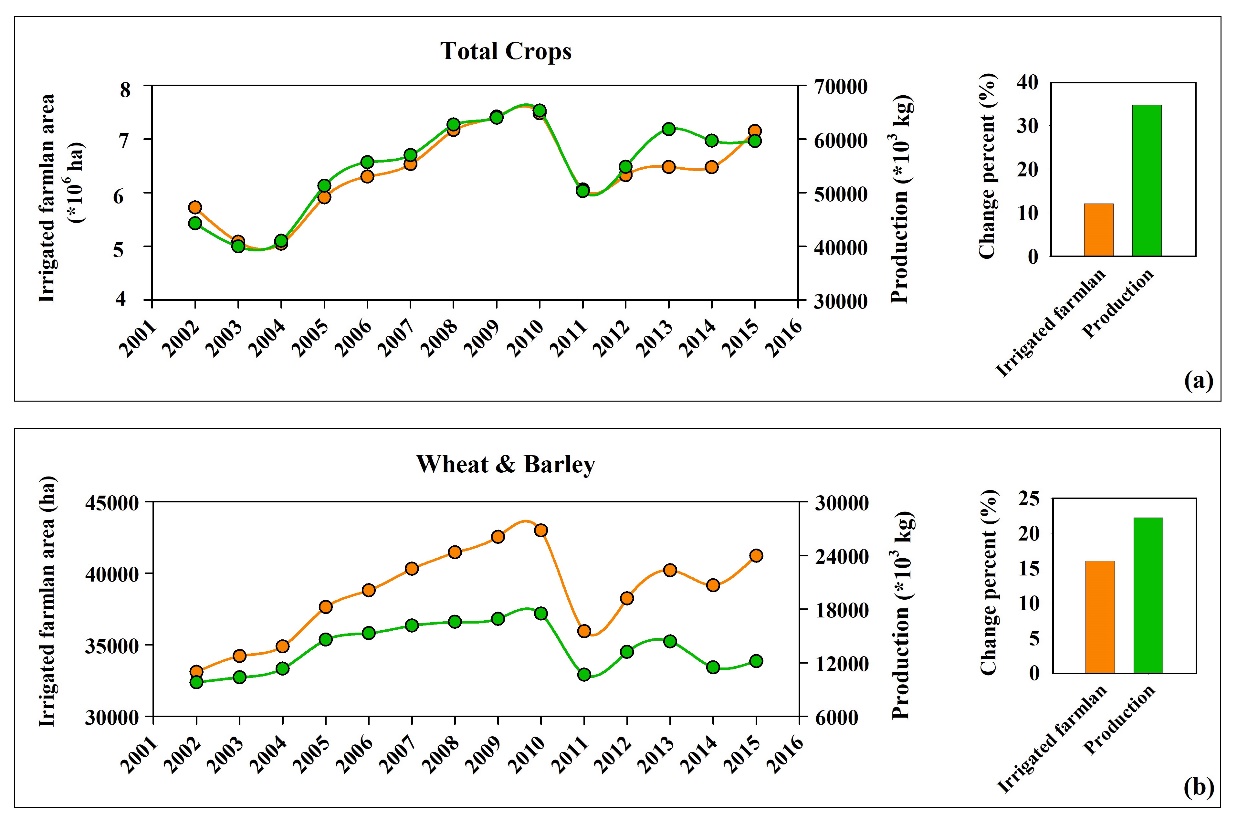


Figure S9. Irrigated farmland and production for (a) total crops and (b) major crops of Iran during 2002 to 2015. (Source of data: Iran’s Ministry of Agriculture Jihad: [https://www.maj.ir/Index.aspx?page_=form&lang=1&PageID=11583&tempname=amar&sub=65&methodName=ShowModuleContent#](https://www.maj.ir/Index.aspx?page_=form&lang=1&PageID=11583&tempname=amar&sub=65&methodName=ShowModuleContent)). (This figure is created using R).


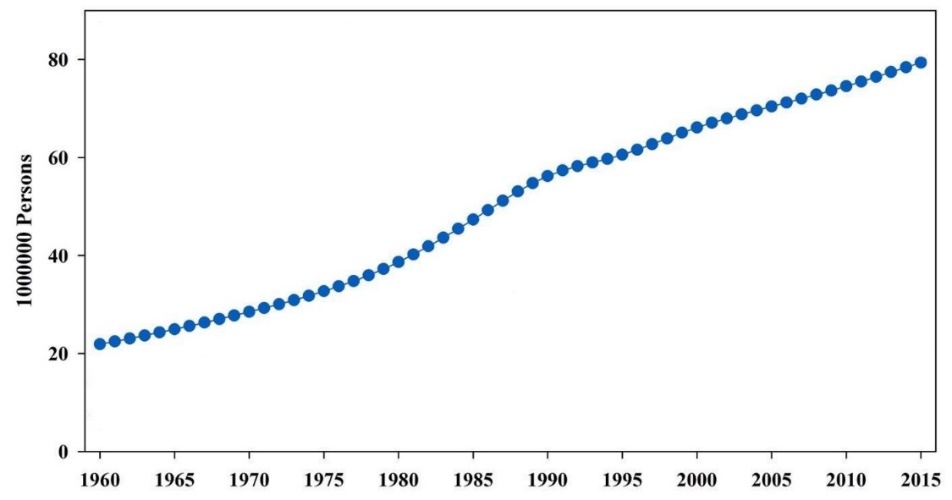


Figure S10. Evolution in Iran’s population from 1960 to 2015 (Source of data: The World Bank: <https://data.worldbank.org/country/iran-islamic-rep>). (This figure is created using R).


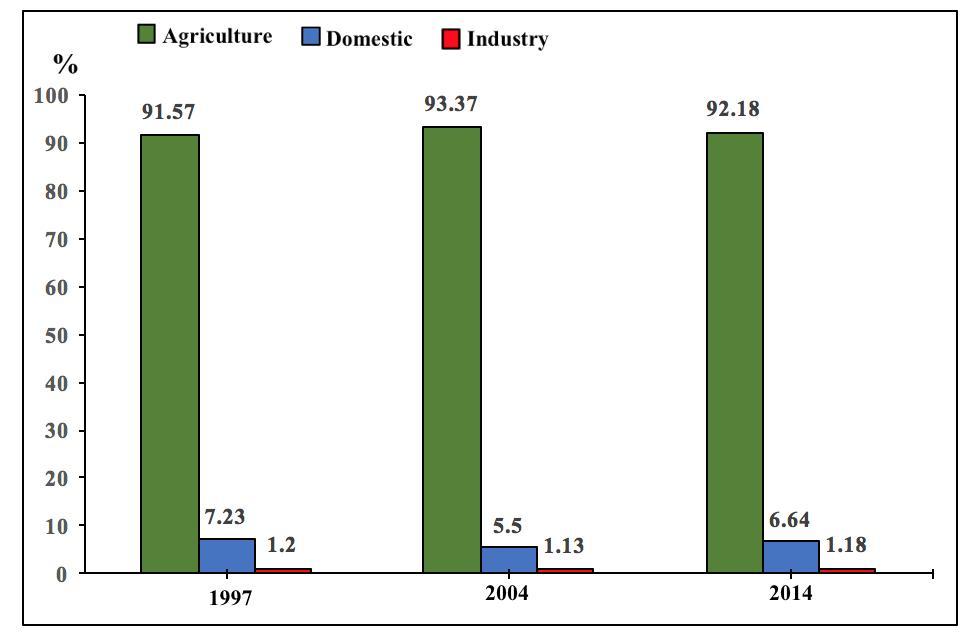


Figure S11. Iran’s annual freshwater withdrawals for agricultural, domestic and industrial uses, based on data gathered from from 1997 to 2014 (Source of data: The World Bank: <http://data.worldbank.org/indicator/ER.H2O.FWAG.ZS?locations=IR>). (This figure is created using R).


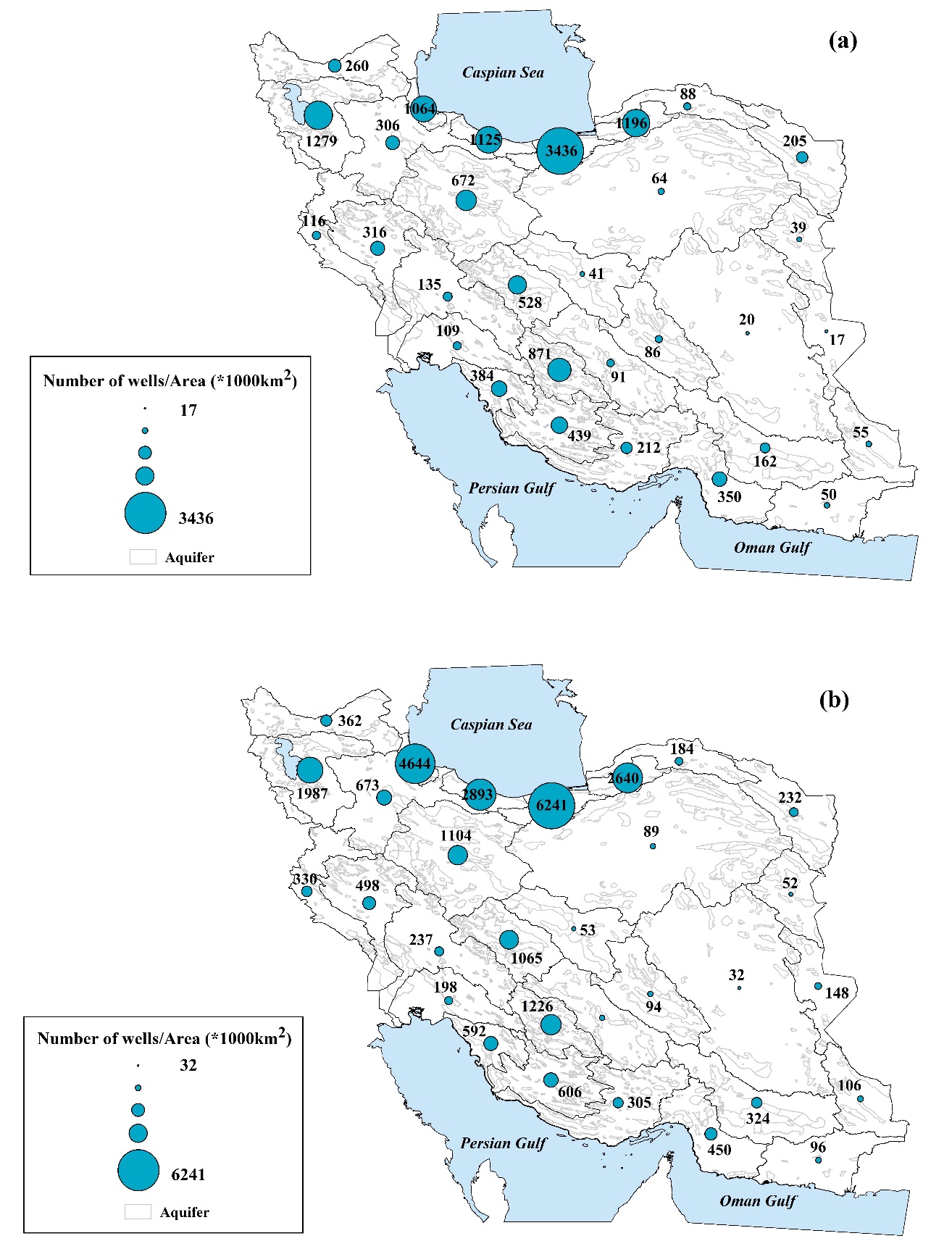


Figure S12. Density of groundwater level monitoring wells at the (a) beginning (2002) and (b) end year (2015) of the study period in each basin across the country (Source of data: Iran’s Ministry of Energy- Iran Water Resources Management Company: <http://wrs.wrm.ir/amar/register.asp>). (This figure is created using ArcGIS 10.8).


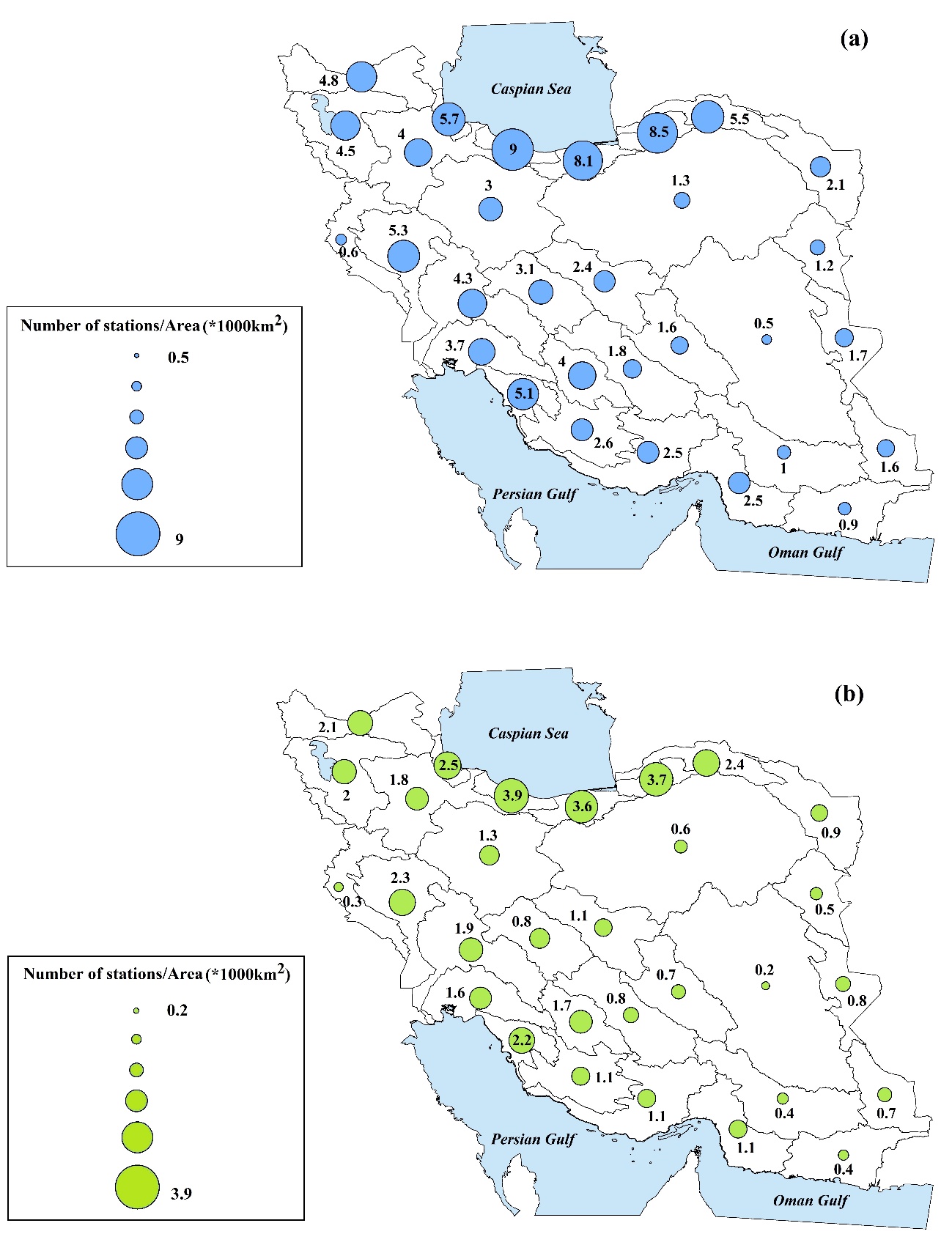


Figure S13. Density of (a) Rain-gage and (b) Evaporation stations in each basin across the country (Source of data: Iran’s Ministry of Energy- Iran Water Resources Management Company: <http://wrs.wrm.ir/amar/register.asp>). (This figure is created using ArcGIS 10.8).
